# Maternal diabetes and childhood cancer risks in offspring: two population-based studies

**DOI:** 10.1038/s41416-022-01961-w

**Published:** 2022-09-10

**Authors:** Xiwen Huang, Johnni Hansen, Pei-Chen Lee, Chia-Kai Wu, Noah Federman, Onyebuchi A. Arah, Chung-Yi Li, Jorn Olsen, Beate Ritz, Julia E. Heck

**Affiliations:** 1 Department of Epidemiology, Fielding School of Public Health, and Jonsson Comprehensive Cancer Center, University of California, Los Angeles (UCLA), Los Angeles, CA 90095-1772 USA; 2 Danish Cancer Society Research Center, Strandboulevarden 49, DK-2100 Copenhagen, Denmark; 3 Department of Health Care Management, National Taipei University of Nursing and Health Sciences, 89 Nei-Chiang St, Wan-Hua Dist, Taipei, 10845 Taiwan; 4 Université Paris-Saclay, UVSQ, Univ. Paris-Sud, Inserm U1018, Team “Exposome, heredity, cancer and health”, CESP, 94807 Villejuif, France; 5 Department of Public Health, National Cheng Kung University, #1, University Road, Tainan, 70101 Taiwan; 6 Department of Pediatrics, Geffen School of Medicine, UCLA, Los Angeles, CA 90095-1752 USA; 7 Department of Statistics, UCLA College of Letters and Science, Los Angeles, CA USA; 8 Section for Epidemiology, Department of Public Health, Faculty of Health, Aarhus University, Aarhus, Denmark; 9 Department of Public Health, College of Public Health, China Medical University, Taichung, Taiwan; 10 Department of Healthcare Administration, College of Medical and Health Science, Asia University, Taichung, Taiwan; 11 Department of Clinical Epidemiology, Aarhus University, Olof Palmes Allé 43-45 8200 Aarhus N, Aarhus, Denmark; 12 College of Health and Public Service, University of North Texas, 1155 Union Circle #305250, Denton, TX 76203-5017 USA

**Keywords:** Gestational diabetes, Risk factors, Paediatric cancer

## Abstract

**Background:**

The effect of maternal diabetes on childhood cancer has not been widely studied.

**Methods:**

We examined this in two population-based studies in Denmark (*N* = 6420 cancer cases, 160,484 controls) and Taiwan (*N* = 2160 cancer cases, 2,076,877 non-cases) using logistic regression and Cox proportional hazard regression adjusted for birth year, child’s sex, maternal age and birth order.

**Results:**

Gestational diabetes in Denmark [odds ratio (OR) = 0.98, 95% confidence interval (CI): 0.71–1.35] or type II and gestational diabetes in Taiwan (type II: hazard ratio (HR) = 0.81, 95% CI: 0.63–1.05; gestational diabetes: HR = 1.06, 95% CI: 0.92–1.22) were not associated with cancer (all types combined). In Denmark, maternal type I diabetes was associated with the risk of glioma (OR = 2.33, 95% CI: 1.04–5.22), while in Taiwan, the risks of glioma (HR = 1.59, 95% CI: 1.01–2.50) were elevated among children whose mothers had gestational diabetes. There was a twofold increased risk for hepatoblastoma with maternal type II diabetes (HR = 2.02, 95% CI: 1.02–4.00).

**Conclusions:**

Our results suggest that maternal diabetes is an important risk factor for certain types of childhood cancers, emphasising the need for effective interventions targeting maternal diabetes to prevent serious health effects in offspring.

## Background

Childhood cancer is a rare disease, but the incidence rates have been slowly rising over the past few decades worldwide [1]. Leukaemia and central nervous system (CNS) tumours are among the most common childhood cancers in developed countries [2]. The incidence of several childhood cancer types peaks in early life and infancy, implicating events prior to conception or during gestation in the genesis of the disease. However, germline mutations are estimated to play a role in less than 10% of all paediatric cancer diagnoses [3]. Prenatal exposure to radiation [4] and diethylstilbestrol [5] are established causes of childhood cancers but are relatively rare.

Women of childbearing age are at increased risk of type I, type II and gestational diabetes internationally [6, 7], driven in part by the increasing prevalence of obesity among pregnant women [8]. In pregnancy, diabetes promotes foetal growth and increases the expression of proinflammatory cytokines in the placenta, presenting possible biologic mechanisms linking these disorders to childhood cancers [9–11]. However, the impact of maternal diabetes on childhood cancer risk has not been extensively studied. A higher risk of leukaemia has previously been associated with all types of maternal diabetes [12–15]. One study differentiated between type I and type II diabetes and reported that maternal type I diabetes but not type II diabetes was associated with a higher risk of leukaemia in offspring [15]. Results for other types of childhood cancer have been inconsistent [12, 13, 15–20].

Maternal gestational diabetes has been known to be related to race/ethnicity, with non-Hispanic whites having the lowest risk and Asians having the highest risk [21]. Studies in the U.S. have consistently demonstrated that women from racial and ethnic minority groups (i.e., Native American, Hispanic, Asian, Black) are more likely to have type I or type II diabetes than are non-Hispanic white women [7, 22]. Using two population-based registries in Denmark and Taiwan, we aimed to examine the contributions of different types of maternal diabetes to the risks of childhood cancers. With access to nationwide registries in Denmark and Taiwan, we were able to investigate the risks of childhood cancers in these populations with distinctly different underlying distributions of maternal diabetes and childhood cancer types. A previous cohort study in Denmark reported increased risks of acute lymphoblastic leukaemia (ALL) (age <15) among offspring of mothers with pregestational and gestational diabetes during the birth years 1996–2015 [23]. Here, we expand the study period back in time (1977–2013) and look across other cancer types up to the age of 19 following exposure to maternal diabetes in utero.

## Methods

### Denmark

We included all cancer cases born in Denmark between 1977 and 2013, aged 0–19 at diagnosis, and diagnosed between 1977 and 2016. Controls, randomly selected from the Central Population Registry [24], were frequency matched by birth year and sex (ratio 1:25) and free of cancer at the date of diagnosis of the corresponding case. Children who were likely not viable (gestational age ≤20 weeks or birthweight <500 g, *n* = 17) were excluded, resulting in 6420 cases and 160,484 controls for the final analyses. This case–control data set has been used previously to study the risk of childhood cancers from occupational and perinatal factors [25–29]. The cases and controls were linked to the Medical Births Registry [30] and Danish National Patient Registry [31] based on their unique Central Person number assigned at birth. This 10-digit number includes information on the date of birth and sex.

Childhood cancer cases were identified from the Danish Cancer Registry that contains information on the Central Person number, morphology, topography, and date of diagnosis, among other factors [32]. The diagnosis was based on the International Classification of Diseases (ICD-O) until 2003, and the International Classification of Diseases, Revision 10 (ICD-10) thereafter; the subtype of cancer was based on morphology recorded in the International Classification of Childhood Cancer (ICCC) revision one until 2003 and revision three thereafter [1, 33]. For CNS tumours, we included both malignant and benign tumours. Astrocytoma, the main subtype of gliomas, was reported separately if the effect estimates were different from the results of gliomas overall. We included in the analysis data where there were more than 5 exposed cases for ALL (ICCC-1: 011-012; ICCC-3: 011), CNS tumours (ICCC-1: 031-036; ICCC-3: 031-036), gliomas (ICD-O-1 and ICD-O-3 histology codes: 9380–9384, 9391–9460) [34].

Information on maternal diabetes diagnoses was retrieved from the Danish National Patient Registry (1977–2016) and the Medical Births Registry (1977–2013) using International Classification of Disease codes (ICD-8 codes during 1970–1993 and ICD-10 codes from 1994). Type I diabetes (ICD-8: 249; ICD-10: E10, O24.0) and type II diabetes (ICD-8: 250; ICD-10: E11, O24.1) were identified for mothers who had received an ICD-8/10 code of diabetes before childbirth. Type I and type II diabetes were defined as a diagnosis before childbirth rather than before pregnancy because preexisting, but undiagnosed, diabetes is likely to be registered during pregnancy, in accordance with previous studies of maternal diabetes in Denmark [35, 36]. Because type I or type II diabetes were recorded using the same code (ICD-8: 250) between 1977 and 1986, the below criterium were used to differentiate between type I and type II diabetes during that period: a specific code of type I or type II diabetes registered later or age at diagnosis of diabetes (type I: < 30 years and type II: ≥30 years) [35, 37, 38]. If a mother was diagnosed with both types before the index childbirth (*n* = 100, 5.1% of diabetes cases), she was classified according to the type that was first diagnosed. Gestational diabetes was identified for mothers who did not have a diagnosis of type I or type II diabetes and had received a diagnosis of gestational diabetes during the index pregnancy (ICD-8: 63474, Y6449; ICD-10: O24.4, O24.9) [37].

Multivariable unconditional logistic regression analyses were used to estimate associations with maternal diabetes and childhood cancer in offspring. All models were adjusted for matching variables, birth year and child’s sex. We utilised unconditional logistic regression, breaking the matching, to improve statistical power [39]. The selection of additional covariates was based on the literature, and we applied the change-in-estimate-criterium (included covariates that changed estimates by 10% or more). For maternal diabetes, we identified potential confounders from previous studies [12, 13, 15] (i.e., maternal age, birth order). We also considered adjustment for other covariates, including paternal age, maternal socioeconomic status [40], maternal smoking at the first prenatal visit (ever vs. never), maternal birthplace (i.e., Denmark, Europe, other), and maternal infections during pregnancy, but adjustment for these factors did not change point estimates by more than 10%. Therefore, the final models adjusted for birth year, child’s sex, maternal age, and birth order. Birthweight, gestational age, or presence of congenital anomalies were not adjusted as we consider these factors to be on the causal pathway, and adjustment for them may cause collider stratification bias [41]. Because it has been suggested that age at diagnosis modifies the effect estimate of maternal diabetes on childhood cancer risk, we additionally examined the associations stratified by age at diagnosis of the offspring (0–14, 15–19 years) for CNS tumours; this also allowed us to compare our results to previous studies [15].

In a sensitivity analysis of the possible cancer risks following maternal diabetes, we additionally adjusted for covariates which were only available for a subset of the overall sample (i.e., maternal smoking during pregnancy (available since 1991), maternal pre-pregnancy BMI (available since 2003). All statistical analyses were conducted using SAS, Version 9.4 (SAS Institute Inc., Cary, NC, USA).

Sample sizes were predetermined by the number of cancers in Denmark. According to our power calculations, a study with the number of cases and controls we selected will allow us to detect an odds ratio (OR) for childhood cancer of 1.5 or more in those prenatally exposed to maternal diabetes compared to those prenatally unexposed to maternal diabetes, at an exposure prevalence of 0.3% in population controls (power = 80%, two-sided *P* value = 0.05).

### Taiwan

The data we relied on have been previously described [42]. In brief, cases were ascertained from the Taiwanese Cancer Registry. Maternal type I (ICD-9: 250.x1, 250.x3) or type II (ICD-9: 250.0–250.9 except 250.x1–250.x3) or gestational diabetes status (ICD-9: 648.0, 648.8) recorded as ICD-9 was acquired through linkage to the National Health Insurance Research Database provided by the Health and Welfare Data Science Center in Taiwan. The current analysis includes 2,160 cancer cases, aged 0–11 at diagnosis between 2004 and 2014 and 2,076,877 non-cases born between 2004 and 2014. We included in the analysis where there were more than 5 exposed cases which included ALL (ICCC-1: 011-012; ICCC-3: 011), acute myeloid leukaemia (ICCC-1: 013; ICCC-3: 012), non-Hodgkin’s lymphoma (ICCC-1: 022, 023; ICCC-3: 022, 023), CNS tumours (ICCC-1: 031-036; ICCC-3: 031-036), gliomas (ICD-O-1 and ICD-O-3 histology codes: 9380–9384, 9391–9460) [34], retinoblastoma (ICCC-1: 050; ICCC-3: 050), medulloblastoma (ICD-O-1 and ICD-O-3 histology codes: 9470-9472) [34], neuroblastoma (ICCC-1: 041; ICCC-3: 041), hepatoblastoma (ICCC-1: 071; ICCC-3: 071), germ cell tumours (ICCC-1: 101-104; ICCC-3: 101-105).

We estimated the associations between maternal diabetes and risks of paediatric cancer types using Cox proportional hazard models to estimate hazard ratios (HRs) and their 95% confidence intervals (CIs). Following the same strategy as in the Danish analyses, we adjusted for the child’s birth year, sex, maternal age and parity in the final model.

## Results

### Denmark

Demographic and gestational characteristics were similar among cases and controls (Supplemental Table 1). Mothers with diabetes tended to be older, less likely to report smoking, and more likely to be born outside of Europe, and the children of diabetic mothers had, as expected, higher birthweight and later birth order (Supplemental Table 2).

Overall, gestational diabetes (OR = 0.98) was not associated with cancer (all types combined) in Denmark. Maternal type I diabetes (OR = 1.34, 95% CI: 0.92–1.94) and type II diabetes (OR = 1.64, 95% CI: 0.97–2.77) appeared to be associated with higher all type cancer risks, although the estimated confidence intervals crossed the null compatible with no risk increase. While the point estimates for ALL in offspring were above one for maternal type I diabetes (OR = 1.44, 95% CI: 0.64–3.23) and gestational diabetes (OR = 1.38, 95% CI: 0.77–2.45; Table 1), the confidence intervals were wide and included the null value. For CNS tumours, we observed a positive association with type I diabetes (OR = 2.44, 95% CI: 1.40–4.24), while there was no association with gestational diabetes (OR = 0.95). Children prenatally exposed to type I diabetes had a higher risk of malignant CNS tumours (7 exposed cases, OR = 2.18, 95% CI: 1.04–4.62) and benign CNS tumours (six exposed cases, OR = 2.81, 95% CI: 1.25–6.31). Gliomas were positively associated with prenatal exposure to maternal type I diabetes (OR = 2.33, 95% CI: 1.04–5.22), and the positive association was stronger for astrocytoma (six exposed cases, OR = 3.61, 95% CI: 1.61–8.11), but less strong and imprecise suggesting no association with gestational diabetes (OR = 1.36, 95% CI: 0.57–3.36). For type II diabetes, we were not able to examine associations with specific types of cancer due to the limited sample size.

**Table 1:** Maternal diabetes and childhood cancer risks in Denmark (birth year ≥1977).

	Total N	N (%)	Crude model	Adjusted model*
			OR (95% CI)	OR (95% CI)
Controls	16,0484			
Type I diabetes		544 (0.3)	Ref.	Ref.
Type II diabetes		229 (0.1)	Ref.	Ref.
Gestational diabetes		1014 (0.6)	Ref.	Ref.
Cases: all cancers	6420			
Type I diabetes		29 (0.5)	1.34 (0.92, 1.94)	1.34 (0.92, 1.94)
Type II diabetes		15 (0.2)	1.62 (0.96, 2.73)	1.64 (0.97, 2.77)
Gestational diabetes		40 (0.6)	0.99 (0.72, 1.36)	0.98 (0.71, 1.35)
ALL	1217			
Type I diabetes		6 (0.5)	1.45 (0.65, 3.25)	1.44 (0.64, 3.23)
Gestational diabetes		12 (1.0)	1.42 (0.80, 2.53)	1.38 (0.77, 2.45)
Central nervous system tumours	1575			
Type I diabetes		13 (0.8)	2.44 (1.41, 4.24)	2.44 (1.40, 4.24)
Gestational diabetes		9 (0.6)	0.94 (0.48, 1.82)	0.95 (0.49, 1.84)
Gliomas	775			
Type I diabetes		6 (0.8)	2.34 (1.04, 5.24)	2.33 (1.04, 5.22)
Gestational diabetes		5 (0.7)	1.36 (0.56, 3.29)	1.38 (0.57, 3.36)

*Ref.* reference, *ALL* acute lymphoblastic leukaemia.

^*^Adjusted for birth year, sex, maternal age, birth order.

Note: cancer types presented here have at least five cases exposed to maternal diabetes.

When we combined all types of diabetes, we saw no increase for all types of cancer combined (OR = 1.16, 95% CI: 0.93–1.45). However, the risk of gliomas in offspring was increased by prenatal exposures to any of the three types of maternal diabetes (OR = 1.76, 95% CI: 1.01–3.06). The point estimate of CNS tumours (OR = 1.45, 95% CI: 0.97–2.16) was elevated, but the confidence intervals were wide and included the null.

The estimated effect of any type of diabetes or maternal type I diabetes on the risk of CNS tumours in offspring varied by age at diagnosis, with diagnosis age 15–19 being most strongly associated with CNS tumours (any diabetes: OR = 3.63, 95% CI: 1.85–7.13; type I: OR = 6.64, 95% CI: 3.08–14.31; Table 2).

**Table 2:** The risk of central nervous system tumours among offspring of mothers with diabetes, stratified by age at diagnosis in Denmark.

	Any diabetes		Type I diabetes	
Age groups of offspring (years)	*N* (%)	Adjusted model* OR (95% CI)	*N* (%)	Adjusted model* OR (95% CI)
0–14	16 (1.3)	1.06 (0.64, 1.74)	6 (0.5)	1.41 (0.63, 3.17)
15–19	9 (2.9)	3.63 (1.85, 7.13)	7 (2.2)	6.64 (3.08, 14.31)

^*^Adjusted for birth year and sex, maternal age, birth order.

Sensitivity analyses with additional adjustment for maternal smoking and pre-pregnancy BMI for diabetes yielded largely similar results as the main findings (Supplemental Tables 3 and 4).

### Taiwan

Cancer cases were more likely born in earlier years with low or high birthweight, in families with lower income and younger parental age, and with higher birth order (Supplemental Table 5). In total, 285,010 (13.9%) children had a mother with a diabetes diagnosis before the index childbirth. The prevalence of maternal diabetes in this population increased from 6.6% in 2004 to 11.8% in 2014. Mothers with diabetes were slightly older and more likely to have higher family income and to live in urban areas. Children whose mothers had diabetes tended to have higher birthweight (Supplemental Table 6).

Overall, gestational diabetes (HR = 1.06) did not elevate the risk of cancer (all types combined) in offspring in Taiwan. When we examined the effect on a specific type of childhood cancer, we found an increased risk of gliomas in offspring prenatally exposed to gestational diabetes compared to unexposed children (HR = 1.59, 95% CI: 1.01–2.50; Table 3). However, no association was observed with type II diabetes (HR = 1.10, 95% CI: 0.49–2.49). Relatively few mothers in Taiwan had a diagnosis of type II diabetes, and few glioma cases were identified in offspring in this subgroup of women. We observed an increased risk of hepatoblastoma in offspring prenatally exposed to maternal type II diabetes compared to the unexposed (HR = 2.02, 95% CI: 1.02–4.00). For other cancer types, including acute myeloid leukaemia, non-Hodgkin’s lymphoma, medulloblastoma, neuroblastoma, and germ cell tumours, we also did not observe an association with type II or gestational diabetes when the sample size allowed us to conduct an analysis. For type I diabetes, we were not able to examine associations with any or specific types of cancer due to the limited sample size.

**Table 3:** Maternal diabetes and childhood cancer risks in Taiwan (2004–2014).

		Type II diabetes			Gestational diabetes		
	Total *N*	*N* (%)	Crude model HR (95% CI)	Adjusted model* HR (95% CI)	*N* (%)	Crude model HR (95% CI)	Adjusted model* HR (95% CI)
Non-cases	2,076,877	80,947 (3.9)	Ref.	Ref.	202,881 (9.8)	Ref.	Ref.
All cancers	2160	64 (3.0)	0.82 (0.64–1.05)	0.81 (0.63–1.05)	211 (9.8)	1.06 (0.92–1.22)	1.06 (0.92–1.22)
ALL	612	19 (3.1)	0.88 (0.56–1.38)	0.88 (0.56–1.39)	64 (10.5)	1.15 (0.89–1.49)	1.16 (0.89–1.51)
AML	155	4 (2.6)	–	–	13 (8.4)	0.90 (0.51–1.59)	0.92 (0.52–1.63)
NHL	438	14 (3.2)	0.93 (0.54–1.58)	0.82 (0.48–1.40)	39 (8.9)	0.98 (0.70–1.36)	0.91 (0.65–1.26)
Central nervous system tumours	293	8 (2.7)	0.77 (0.38–1.55)	0.78 (0.39–1.59)	35 (12.0)	1.33 (0.94–1.90)	1.38 (0.96–1.96)
Gliomas	169	6 (3.6)	1.03 (0.45–2.32)	1.10 (0.49–2.49)	22 (13.0)	1.49 (0.95–2.33)	1.59 (1.01–2.50)
Retinoblastoma	129	≤3	–	–	18 (14.0)	1.52 (0.92–2.50)	1.48 (0.90–2.44)
Medulloblastoma	69	≤3	–	–	6 (8.7)	0.95 (0.41–2.19)	0.96 (0.41–2.22)
Neuroblastoma	226	9 (4.0)	1.06 (0.54–2.06)	1.00 (0.51–1.96)	20 (8.9)	0.92 (0.58–1.44)	0.89 (0.56–1.41)
Hepatoblastoma	113	9 (8.0)	2.18 (1.10–4.30)	2.02 (1.02–4.00)	14 (12.4)	1.32 (0.75–2.30)	1.26 (0.72–2.20)
Germ cell tumours	210	5 (2.4)	0.66 (0.27–1.61)	0.65 (0.27–1.59)	14 (6.7)	0.71 (0.41–1.21)	0.70 (0.41–1.21)

*Ref.* reference, *ALL* acute lymphoblastic leukaemia, *AML* acute myeloid leukaemia, *NHL* non-Hodgkin’s lymphoma.

*Adjusted for birth year and sex, maternal age, parity.

Analysis with additional adjustment for maternal smoking resulted in largely similar results (Supplemental Table 7).

## Discussion

Relying on two population-based cancer registries covering several decades in Denmark and Taiwan, we assessed associations between maternal diabetes and childhood cancers. This is the first study to report on the risk of childhood cancer due to maternal diabetes in Taiwan. The distributions of types of diabetes were very different in the two populations, with type I diabetes being much more common in Denmark and type II and gestational diabetes more common in Taiwan, affecting our statistical power to estimate effects. To our knowledge, the effect that maternal diabetes has on the risk of gliomas among offspring has not been reported previously. Notably, in both Denmark and Taiwan, we observed increases in the risk of offspring developing gliomas, although this was related to different subtypes of diabetes. Thus, the increased risk for gliomas across different diabetes subtypes suggests that the mechanism of action may be related to foetal growth or the intrauterine environment rather than being solely related to autoimmune mechanisms in type I diabetes. Children prenatally exposed to other autoimmune diseases were not observed to have an increased risk of all childhood cancers combined, as reported previously [43].

For gliomas, we observed a positive association with type I and gestational diabetes. Although increased risks were observed, the percentages of gliomas in offspring of women with any type of diabetes were low, with 0.6% and 0.09% among children in Denmark and Taiwan, respectively. Thus, our results between maternal type II diabetes and gliomas are limited by sample size. Results for maternal pregestational diabetes, gestational diabetes, and CNS tumours among offspring have previously been mixed [13, 15]. Our results for CNS tumours were in line with a Finnish population-based registry study that reported a 1.37-fold increased CNS risk in offspring of women with pregestational diabetes, and 1.19-fold increased CNS risk with gestational diabetes, both reported with some statistical uncertainty [13]. Unlike this study, information was not available for the type of pregestational diabetes. Contrary to this, a population-based cohort study in Sweden [15] showed the risk of childhood brain tumours to be reduced with maternal type I diabetes and gestational diabetes for cases diagnosed before age 15. However, when cases diagnosed up to age 20 were included in their analysis, the negative association for maternal diabetes was attenuated and had wide confidence intervals. In our study, the associations between maternal diabetes (i.e., any types of diabetes, type I diabetes) and CNS tumours in offspring were stronger in children with age at diagnosis between 15 and 19 years than for those with age at diagnosis less than 15 years. This may be due to differences in the distribution of CNS tumours such as tumour behaviour, primary site, and histology groups across age groups [44].

For retinoblastoma, our findings support the observation of a previous study that suggested a positive association between gestational diabetes and unilateral retinoblastoma in offspring with wide confidence intervals [16]. In contrast, no association of retinoblastoma with pregestational diabetes was reported in a study in California [45]. However, this study had limited statistical power, and underreporting of maternal diabetes on birth certificates in California is of concern.

In Taiwan, we observed an increase in the risk of hepatoblastoma among children prenatally exposed to type II diabetes. Similar to other regions in Asia [42], Taiwan exhibits a high rate of hepatoblastoma in comparison to Europe [46] and the United States [47], which enables us to investigate this cancer type in children in Taiwan. We had insufficient statistical power to examine this in the Danish analysis. Few studies reported on maternal diabetes and the risk of hepatoblastoma in children. In a study that observed elevated risks with type I or type II diabetes, the confidence interval was wide [12]. Prior associations with gestational diabetes were inconsistent [12, 48].

In our study, the increased risk for gliomas among children prenatally exposed to maternal diabetes in both populations suggests that factors that drive growth in utero may increase the risk of CNS tumours. In addition to the potential mechanism of action through birthweight, a recent meta-analysis found that gestational diabetes was consistently associated with higher maternal insulin-like growth factor-1 (IGF-I) concentrations [49]. In a prospective longitudinal study, IGF-1 concentrations were positively related to subsequent risk of gestational diabetes [50]. It has been shown that high maternal IGF-1 is consistently associated with foetal and placental growth and birthweight [51–53]. IGF-1 has been strongly suggested to be involved in the pathogenesis of gliomas [54].

The main strength of these studies is the reliance on population-based record data, avoiding the possibility of recall bias and minimising selection bias. The relatively large sample sizes provided an opportunity to study subtypes of childhood cancer and allowed us to check and adjust for a number of covariates. This study was still limited by the small number of events resulting in imprecise effect estimates. Cancer types involving <10 exposed cases, as well as those with wide 95% confidence intervals, should be interpreted cautiously. Another limitation was the different prevalence of diabetes types across the populations, which limits comparability between the studies. In comparison to another population-based study in Denmark [36], maternal pregestational diabetes was underreported in our data. This is likely in part due to the underdiagnosis of type I and II diabetes. This could lead to less precision in our effect estimates. In Taiwan, diabetes prevalence was 0.1% and 3.9% for type I and type II diabetes before childbirth between 2004 and 2014, respectively; this is comparable to what has been reported in other population-based studies in Taiwan [55, 56]. Based on previous validation studies, high positive predictive values were observed for ICD diagnoses of type I, type II and gestational diabetes in Denmark [57] and Taiwan [58]. However, we are aware that the more severe form of diabetes has a higher likelihood of being recorded in registers [59]. Furthermore, lower sensitivity when using information from the National Patient Register in Denmark alone to identify gestational diabetes may have caused non-differential exposure misclassification and resulted in an underestimation of the effect [60]. Finally, we did not have access to blood glucose measurements during pregnancy to determine whether glucose levels were adequately controlled. However, a previous study reported the validity of such measurements to be relatively low in Denmark [61]. In our data, birthweight was highest in children whose mothers had type II or gestational diabetes, suggesting that glucose levels were less than optimal in mothers with these types of diabetes. However, there is no evidence to suggest that glucose control during pregnancy modifies the risk of childhood cancer.

In conclusion, maternal diabetes increased the risk of certain childhood cancer in Denmark and Taiwan. Our study supports the potential role for maternal diabetes in cancer risk in offspring and implicates the importance of maintaining normal blood glucose levels to prevent rare but serious adverse health outcomes in the offspring.

## Supplementary information

Supplementary files

## Data Availability

Not applicable.

## Code availability

Statistical coding can be obtained via the corresponding author. All statistical analyses were undertaken using SAS 9.4.
