# Supplementary material for: Maternal diabetes and childhood cancer risks in offspring: two population-based studies
Source: Br J Cancer. 2022 Sep 10;127(10):1837–42. doi: 10.1038/s41416-022-01961-w (PMC9643384; doi:10.1038/s41416-022-01961-w)
Supplement: Supplementary file 1 — Supplementary files [file 41416_2022_1961_MOESM1_ESM.pdf]

Supplemental Table 1. Demographic characteristics of study participants in Denmark, by case control status, 1977-2013 (n=166,904)

	Cases (N = 6,420)	Controls (N = 160,484)
	N (%)	N (%)
Mother's diabetes type		
Type 1 diabetes	29 (0.5)	544 (0.3)
Type 2 diabetes	15 (0.2)	229 (0.1)
GDM	40 (0.6)	1014 (0.6)
Mother pre-pregnancy BMI		
Mean (SD)	24.4 (5.5)	24.1 (4.8)
18.4 or less	34 (4.2)	821 (4.1)
18.5-25	497 (61.1)	12924 (64.4)
25-30	179 (22.0)	4043 (20.1)
30 +	103 (12.7)	2279 (11.4)
Maternal age		
Mean (SD)	28.4 (5.0)	28.3 (5.0)
=<24	1423 (22.2)	36923 (23.0)
25-29	2472 (38.5)	60759 (37.9)
30-34	1744 (27.2)	44546 (27.8)
35-39	669 (10.4)	15695 (9.8)
40+	112 (1.7)	2561 (1.6)
Paternal age		
Mean (SD)	31.2 (5.8)	31.1 (5.8)
=<24	661 (10.4)	17979 (11.3)
25-29	2047 (32.1)	50022 (31.3)
30-34	2050 (32.1)	52045 (32.6)
35-39	1096 (17.2)	26766 (16.8)
40+	526 (8.2)	12842 (8.0)
Family SES		
Academics and high level self-employed	621 (13.3)	15176 (13.0)
Middle-long education	807 (17.3)	19178 (16.4)
Shorter education	879 (18.8)	22312 (19.0)
Skilled worker	1512 (32.4)	38330 (32.7)
Unskilled worker	846 (18.1)	22187 (18.9)
Birth order		
1	2827 (44.0)	68852 (42.9)
2	2400 (37.4)	60938 (38.0)
3 or more	1193 (18.6)	30694 (19.1)
Maternal smoking at the first prenatal visit	818 (23.9)	20753 (24.2)

Child's birthweight (g)		
Mean (SD)	3466.5 (607.9)	3437.8 (585.8)
Extremely low (<1000g)	48 (0.8)	944 (0.6)
Low (1000-2500g)	274 (4.3)	6947 (4.3)
Medium (2500g-4000g)	4846 (75.9)	125336 (78.4)
High (>4000g)	1219 (19.1)	26606 (16.6)
Maternal birthplace		
Denmark	5853 (91.3)	146777 (91.6)
Other Europe	217 (3.4)	5523 (3.4)
Other	338 (5.3)	7893 (4.9)
Urban or rural birthplace		
Urban	2114 (32.9)	50981 (31.8)
Small towns	1808 (28.2)	46632 (29.1)
Rural	2498 (38.9)	62871 (39.2)

SES: Socioeconomic status.

GDM: Gestational diabetes mellitus

Supplemental Table 2. Demographic characteristics of study participants, stratified by any type of maternal diabetes status, in Denmark, 1977-2013 (N = 166,904)

	Children with mothers who had diabetes (N = 1,948)	Children with mothers who did not have diabetes (N = 164,956)
	N (%)	N (%)
Mother pre-pregnancy BMI	28.6 (6.3)	24.0 (4.7)
18.4 or less	10 (1.6)	845 (4.2)
18.5-25	191 (29.8)	13230 (65.4)
25-30	202 (31.5)	4020 (19.9)
30 +	239 (37.2)	2143 (10.6)
Child’s sex		
Male	1047 (53.7)	90340 (54.8)
Female	901 (46.3)	74616 (45.2)
Maternal age		
Mean (SD)	30.9 (5.2)	28.3 (4.9)
=<24	219 (11.2)	38127 (23.1)
25-29	618 (31.7)	62613 (38.0)
30-34	598 (30.7)	45692 (27.7)
35-39	397 (20.4)	15967 (9.7)
40+	116 (6.0)	2557 (1.6)
Paternal age		
Mean (SD)	33.5 (6.4)	31.0 (5.8)
=<24	97 (5)	18543 (11.3)
25-29	458 (23.6)	51611 (31.5)
30-34	597 (30.8)	53498 (32.6)
35-39	477 (24.6)	27385 (16.7)
40+	311 (16)	13057 (8)
Family SES		
Academics and high level self-employed	108 (9.1)	15689 (13.0)
Middle-long education	172 (14.5)	19813 (16.4)
Shorter education	232 (19.6)	22959 (19.0)
Skilled worker	403 (34.0)	39439 (32.7)
Unskilled worker	270 (22.8)	22763 (18.9)
Birth order		
1	669 (34.3)	71010 (43.0)
2	800 (41.1)	62538 (37.9)
3 or more	479 (24.6)	31408 (19.0)
Maternal smoking at the first prenatal visit	303 (21.3)	21268 (24.2)
Child's birthweight (g)		

Mean (SD)	3512.6 (684.7)	3438.0 (585.4)
Extremely low (<1000g)	13 (0.7)	979 (0.6)
Low (1000-2500g)	122 (6.3)	7099 (4.3)
Medium (2500g-4000g)	1339 (68.9)	128843 (78.4)
High (>4000g)	468 (24.1)	27357 (16.7)
Maternal birthplace		
Denmark	1661 (85.4)	150969 (91.7)
Other Europe	62 (3.2)	5678 (3.4)
Other	222 (11.4)	8009 (4.9)
Urban or rural birthplace		
Urban	688 (35.3)	52407 (31.8)
Small towns	489 (25.1)	47951 (29.1)
Rural	771 (39.6)	64598 (39.2)

SES: Socioeconomic status

SD: Standard deviation

Supplemental Table 3. Sensitivity analysis examining the associations of type I diabetes and gestational diabetes with childhood cancer risks in Denmark (1991+, N = 89,232)

	Type I Diabetes			
	Total N	N (%) with type I diabetes	*Odds Ratio (95% Confidence Interval)	**Odds Ratio (95% Confidence Interval)
Controls	85813	289 (0.3)	Ref.	Ref.
All cancers	3419	14 (0.4)	1.21 (0.71, 2.08)	1.21 (0.71, 2.08)
ALL	682	5 (0.7)	2.14 (0.88, 5.19)	2.14 (0.88, 5.20)
Central nervous system	812	7 (0.9)	2.60 (1.22, 5.52)	2.60 (1.22, 5.52)
Gliomas	341	5 (1.5)	4.51 (1.85, 11.0)	4.51 (1.85, 11.0)
	Gestational Diabetes			
	Total N	N (%) with gestational diabetes	*Odds Ratio (95% Confidence Interval)	**Odds Ratio (95% Confidence Interval)
Controls	85813	908 (1.1)	Ref.	Ref.
All cancers	3419	38 (1.1)	1.05 (0.76, 1.46)	1.05 (0.76, 1.46)
ALL	682	12 (1.8)	1.49 (0.83, 2.65)	1.49 (0.83, 2.65)
Central nervous system	812	8 (1.0)	0.96 (0.47, 1.93)	0.95 (0.47, 1.93)
Gliomas	341	< 5	-----	-----

*Adjusted for birth year and sex, maternal age, birth order

** Additionally adjusted for maternal smoking

Ref.: Reference

ALL: Acute lymphoblastic leukemia

Supplemental Table 4. Sensitivity analysis examining the associations of pregestational diabetes and gestational diabetes with childhood cancer risks in Denmark (2003+, N = 20,880)

	Pregestational Diabetes			
	Total N	N (%) with pregestational diabetes	*Odds Ratio (95% Confidence Interval)	**Odds Ratio (95% Confidence Interval)
Controls	2006	149 (0.7)	Ref.	Ref.
All cancers	813	7 (0.9)	1.17 (0.54, 2.50)	1.13 (0.53, 2.42)
ALL	201	< 5	----	----
Central nervous system	186	< 5	----	----
Gliomas	64	< 5	----	----
	Gestational Diabetes			
	Total N	N (%) with gestational diabetes	*Odds Ratio (95% Confidence Interval)	**Odds Ratio (95% Confidence Interval)
Controls	2006	465 (2.3)	Ref.	Ref.
All cancers	813	21 (2.6)	1.12 (0.72, 1.75)	1.05 (0.67, 1.64)
ALL	201	8 (4.0)	1.70 (0.83, 3.49)	1.74 (0.84, 3.61)
Central nervous system	186	< 5	----	----
Gliomas	64	< 5	----	----

*Adjusted for birth year and sex, maternal age, birth order

** Additionally adjusted for pre-pregnancy BMI

Ref.: Reference

ALL: Acute lymphoblastic leukemia

Note: Due to limited sample size, we were not able to examine the change in the estimates with and without adjustment of pre-pregnancy BMI for the association between maternal type I diabetes and any subtype or all types of childhood cancer, so the results presented here combined all types of pregestational diabetes.

Supplemental Table 5. Demographic characteristics of cancer cases and controls in Taiwan, birth years 2004-2014

	Cancer Cases<br>N=2,160<br>N (%)	Non-cases<br>N=2,076,877<br>N (%)
Maternal diabetes		
Type I diabetes	< 3	2372(0.1)
Type II diabetes	64 (3.0)	80947(3.9)
Gestational diabetes	211 (9.8)	202881(9.8)
Birth year		
2004	291 (13.5)	199381 (9.6)
2005	297 (13.8)	191774 (9.2)
2006	249 (11.5)	190892 (9.2)
2007	252 (11.7)	192909 (9.3)
2008	222 (10.1)	187788 (9.03)
2009	230 (10.6)	185401 (8.93)
2010	141 (6.5)	151020 (7.3)
2011	155 (7.2)	182461 (8.8)
2012	156 (7.2)	205961 (9.9)
2013	92 (4.3)	191559 (9.2)
2014	75 (3.5)	197731 (9.5)
Maternal age		
Mean (SD)	30.0 (4.8)	30.2 (4.8)
<20	41 (1.9)	31044 (1.5)
20-29	956 (44.3)	863624 (41.6)
30-34	804 (37.2)	797993 (38.4)
35-39	291 (13.5)	331300 (16.0)
40+	68 (3.2)	52916 (2.6)
Maternal history of cancer		
Yes	43 (2.0)	31809 (1.5)
Paternal age		
Mean (SD)	33.1 (5.5)	33.2 (5.3)
<20	5 (0.2)	3485 (0.2)
20-29	510 (23.6)	454359 (21.9)
30-34	779 (36.1)	760634 (36.6)
35-39	479 (22.2)	511717 (24.6)
40+	242 (11.2)	220401 (10.6)
Missing	145 (6.7)	126281 (6.1)

Paternal history of cancer		
Yes	34 (1.6)	22987(1.1)
Family income (New Taiwan dollar)		
<26400	554 (25.7)	498013 (24.0)
26400-45600	542 (25.1)	497747 (24.0)
45600-70850	488 (22.6)	500029 (24.1)
≥70850	485 (22.5)	498801(24.0)
Missing	91 (4.2)	82287 (4.0)
Urbanization level of inhabited area		
High	1078 (50.0)	1102672 (53.1)
Middle	876 (40.6)	781604 (37.6)
Low	206 (9.5)	192601 (9.3)
Mother's birthplace		
Taiwan	1302 (60.3)	1400491 (67.4)
Foreign born	141 (6.5)	132170 (6.4)
Missing	717 (33.2)	544216 (26.2)
Sex		
Male	1197 (55.4)	1079059 (52.0)
Female	963 (44.6)	997718 (48.0)
Missing	0 (0.0)	100 (7.5)
Birth weight (g)		
Mean (SD)	3070.6 (485.6)	3078.1 (449.5)
<2500	185 (8.6)	156525 (7.5)
2500-3999	1926 (89.2)	1882670 (90.7)
≥4000	49 (2.3)	37682 (1.8)
Gestational age (weeks)		
Mean (SD)	38.1 (1.9)	38.3 (1.7)
Very preterm (<33)	45 (2.1)	23053 (1.1)
Preterm (33-36)	199 (9.2)	160438 (7.7)
Term (≥37)	1916 (88.7)	1893386 (91.2)
Size for gestational age		
Lowest 10%	185 (8.6)	195988 (9.4)
Middle 80%	1785 (82.6)	1712398 (82.5)
Highest 10%	190 (8.8)	168491 (8.1)
Method of delivery		
Vaginal	1355 (62.7)	1331991 (64.1)
Cesarean section	805 (37.3)	744886 (35.9)
Multiple birth		

Singleton	2087 (96.6)	2015039 (97.0)
Twin or more	73 (3.4)	61838 (3.0)
Parity		
1	832 (38.5)	807516 (38.9)
2	1010 (46.8)	1044027 (50.3)
3 or more	318 (14.7)	225334 (10.9)

SD: Standard deviation

Supplemental Table 6. Demographic characteristics and maternal diabetes in Taiwan (n= 2,079,037)

	Cases (n=2,160)			Non-cases(n=2,076,877)		
	Children of mothers with maternal diabetes (n=276)	Children of mothers without maternal diabetes (n=1,867)	Missing (n=17)	Children of mothers with maternal diabetes (n=284,734)	Children of mothers without maternal diabetes (n=1,769,535)	Missing (n=22,608)
	N (%)	N (%)		N (%)	N (%)	
Birth year						
2004	24(8.7)	264(14.1)	<= 3	18885(6.6)	177494(10.0)	3002
2005	35(12.7)	258(13.8)	4	20156(7.1)	169233(9.6)	2385
2006	32(11.6)	214(11.5)	<= 3	21818(7.7)	166947(9.4)	2127
2007	30(10.9)	219(11.7)	<= 3	24163(8.5)	166520(9.4)	2226
2008	30(10.9)	188(10.1)	<= 3	24395(8.6)	161327(9.1)	2066
2009	26(9.42)	204(10.9)	<= 3	26365(9.3)	157109(8.9)	1927
2010	16(5.8)	125(6.7)	<= 3	22684(8.0)	126698(7.2)	1638
2011	27(9.8)	128(6.9)	<= 3	28542(10.0)	152215(8.6)	1704
2012	22(8.0)	134(7.2)	<= 3	32692(11.5)	171488(9.7)	1781
2013	20(7.3)	72(3.9)	<= 3	31469(11.1)	158250(8.9)	1840
2014	14(5.1)	61(3.3)	<= 3	33565(11.8)	162254(9.2)	1912
Child's sex						
Male	146(52.9)	1047(56.1)	4	149565(52.5)	917656(51.9)	11838
Female	130(47.1)	820(43.9)	13	135156(47.5)	851792(48.1)	10770
missing	<= 3	<= 3	<= 3	13	87	<= 3
Maternal age						
Mean (SD)	31.3(4.6)	29.8(4.9)	17	31.5(4.6)	30.0(4.8)	22608
<20	<= 3	38(2.0)	<= 3	1728(0.6)	29202(1.7)	114
20-29	94(34.1)	854(45.7)	8	89330(31.4)	761712(43.1)	12582
30-34	112(40.6)	683(36.6)	9	120620(42.4)	670635(37.9)	6738
35-39	55(19.9)	236(12.6)	<= 3	61402(21.6)	267143(15.1)	2755
40+	12(4.4)	56(3.0)	<= 3	11654(4.1)	40843(2.3)	419

Paternal age						
Mean (SD)	34.1(5.0)	32.9(5.5)	17	34.1(5.2)	33.1(5.3)	22608
<20	<= 3	5(0.3)	<= 3	182(0.1)	3298(0.2)	5
20-29	44(16.8)	462(26.6)	4	47880(17.6)	403843(24.4)	2636
30-34	93(35.5)	679(39.1)	9	105548(38.8)	648175(39.1)	6911
35-39	85(32.4)	392(22.6)		81619(30.0)	423297(25.6)	6801
40+	40(15.3)	198(11.4)	4	36717(13.5)	177940(10.7)	5744
Missing	14	131	<= 3	12788	112982	511
Family income (New Taiwan dollar)						
<26400	55(20.5)	488(27.4)	11	53648(19.4)	428844(25.3)	15521
26400-45600	67(25.0)	471(26.4)	6	65789(23.8)	428133(25.3)	3825
45600-70850	63(23.5)	423(23.7)		73012(26.4)	424827(25.1)	2190
≥70850	83(31.0)	402(22.5)	<= 3	84075(30.4)	413936(24.4)	790
Missing	8	83	<= 3	8210	73795	282
Urbanization level of inhabited area						
High	150(54.4)	918(49.2)	10	164073(57.6)	926023(52.3)	12576
Middle	97(35.1)	772(41.4)	7	93242(32.8)	680369(38.5)	7993
Low	29(10.5)	177(9.5)	<= 3	27419(9.6)	163143(9.2)	2039
Parity						
1	107(38.8)	716(38.4)	9	112315(39.5)	681598(38.5)	13603
2	130(47.1)	872(4.67)	8	145842(51.2)	890263(50.3)	7922
3 or more	39(14.1)	279(14.9)	<= 3	26577(9.3)	197674(11.2)	1083
Maternal smoking (before birth)						
Yes	<= 3	8(0.4)	<= 3	2319(0.8)	11478(0.7)	<= 3
No	276(100.0)	1859(99.6)	<= 3	282415(99.2)	1758057(99.4)	437
Missing	<= 3	<= 3	17	<= 3	<= 3	22171
Child's birthweight						
Mean (SD)	3128.7(468.6)	3061.4(488.7)	17	3116.3(466.6)	3070.8(446.5)	22608
Extremely low (<1000g)		11(0.6)	<= 3	350(0.1)	4188(0.2)	36
	17(6.2)					
Low (1000-2500g)		175(9.4)	<= 3	22042(7.7)	143564(8.1)	1218

Medium (2501g-4000g)	249(90.2)	1650(88.4)	17	254864(89.5)	1596961(90.3)	20869
High (>4000g)	10(3.6)	31(1.7)	<= 3	7478(2.6)	24822(1.4)	485
Mother's diabetes type						
Type 1 diabetes	65(23.6)	--------	<= 3	2372(0.8)	--------	<= 3
Type 2 diabetes		--------	<= 3	80947(28.4)	--------	<= 3
GDM	211(76.5)	--------	<= 3	202881(71.3)	--------	<= 3

GDM: gestational diabetes mellitus

Supplemental Table 7. Sensitivity analysis examining the associations of type II diabetes and gestational diabetes with childhood cancer risks in Taiwan (2004-2014)

		**Type II Diabetes**			**Gestational Diabetes**		
	Total N	N (%)	Crude HR (95% CI)	*Adjusted Model HR (95% CI)	N (%)	Crude HR (95% CI)	*Adjusted Model HR (95% CI)
Non-cases	2076877	80947(3.9)	Ref.	Ref.	202881(9.8)	Ref.	Ref.
All cancers	2160	64(3.0)	0.82(0.64-1.05)	0.82(0.64-1.05)	211(9.8)	1.06(0.92-1.22)	1.06(0.92-1.22)
ALL	612	19(3.1)	0.88(0.56-1.38)	0.88(0.56-1.40)	64(10.5)	1.15(0.89-1.49)	1.16(0.89-1.51)
AML	155	4(2.6)	----------	----------	13(8.4)	0.90(0.51-1.59)	0.92(0.52-1.63)
NHL	438	14(3.2)	0.93(0.54-1.58)	0.82(0.48-1.40)	39(8.9)	0.98(0.70-1.36)	0.91(0.65-1.26)
Central nervous system tumors	293	8(2.7)	0.77(0.38-1.55)	0.79(0.39-1.59)	35(12.0)	1.33(0.94-1.90)	1.38(0.96-1.96)
Gliomas	169	6(3.6)	1.03(0.45-2.32)	1.10(0.49-2.49)	22(13.0)	1.49(0.95-2.33)	1.59(1.01-2.50)
Retinoblastoma	129	<=3	----------	----------	18(14.0)	1.52(0.92-2.50)	1.48(0.90-2.44)
Medulloblastoma	69	<=3	----------	----------	6(8.7)	0.95(0.41-2.19)	0.96(0.41-2.22)
Neuroblastoma	226	9(4.0)	1.06(0.54-2.06)	1.00(0.51-1.96)	20(8.9)	0.92(0.58-1.44)	0.89(0.56-1.41)
Hepatoblastoma	113	9(8.0)	2.18(1.10-4.30)	2.02(1.02-4.00)	14(12.4)	1.32(0.75-2.30)	1.26(0.72-2.20)
Germ cell tumors	210	5(2.4)	0.66(0.27-1.61)	0.66(0.27-1.59)	14(6.7)	0.71(0.41-1.21)	0.70(0.41-1.21)

* Adjusted for birth year and sex, maternal age, parity, maternal smoking.
Ref.: Reference
ALL: Acute lymphoblastic leukemia
AML: Acute myeloid leukemia
NHL: Non-Hodgkin's lymphoma
